# The influence of obesity on functional outcomes and patient satisfaction 8 weeks after total knee arthroplasty: results of the prospective FInGK study

**DOI:** 10.1186/s12891-022-05874-w

**Published:** 2022-11-03

**Authors:** Gesa Baum, Hannes Jacobs, Djordje Lazovic, Uwe Maus, Falk Hoffmann, Gesine H. Seeber

**Affiliations:** 1 Department of Health Services Research, Carl von Ossietzky University Oldenburg, Ammerländer Heerstr. 114-118, 26129 Oldenburg, Germany; 2 University Hospital for Orthopaedics and Trauma Surgery Pius-Hospital, Medical Campus University of Oldenburg, Oldenburg, Germany; 3 Department of Orthopaedics and Trauma Surgery, University Hospital Düsseldorf, Düsseldorf, Germany; 4 Department of Orthopaedics, University of Groningen, University Medical Center Groningen, Groningen, The Netherlands

**Keywords:** Knee osteoarthritis, Total knee arthroplasty, Obesity, Patient-reported outcomes, Western Ontario and Mc Masters Universities Osteoarthritis Index, Patient satisfaction

## Abstract

**Objectives:**

To investigate obese versus non-obese subjects´ knee joint function, stiffness, pain, expectations, and outcome satisfaction before and two months after total knee arthroplasty (TKA).

**Methods:**

This study is a secondary analysis of data retrieved via a prospective single-centre cohort study investigating knee joint function and health care services utilization in patients undergoing TKA (FInGK Study). For the primary study, elective TKA patients were consecutively recruited between December 2019 and May 2021. Preoperative expectations, Western Ontario and McMasters Universities Osteoarthritis Index (WOMAC), surgery outcome satisfaction, and sociodemographic variables were assessed via self-reported questionnaires. In the current study, obese (Body Mass Index (BMI) ≥ 30 kg/m²) versus non-obese (BMI < 30 kg/m²) subjects’ data were exploratively compared before and two months after TKA. Multivariable logistic regression assessed factors associated with TKA satisfaction two months postoperatively. Linear regression evaluated factors associated with higher WOMAC change two months postoperatively.

**Results:**

A total of 241 subjects participated (response: 85.2%). Eighty-seven were non-obese (mean age: 70.7 years, 63.2% female) and 154 were obese (mean age: 67.1 years, 57.8% female). Obese subjects reported inferior pre- and postoperative pain and knee joint function compared to non-obese subjects. Yet, WOMAC scores of obese and non-obese subjects significantly improved from preoperative means of 52.6 and 46.8 to 32.3 and 24.4 after surgery, respectively. The only significant TKA satisfaction predictor was subjects’ smoking status. Non-obesity and worse preoperative WOMAC scores were predictive of higher WOMAC change scores after two months.

**Conclusion:**

Both obese and non-obese subjects reported significant symptom improvements. However, as obese subjects’ short-term outcomes were still inferior, more research on TKA rehabilitation measures adapted to the needs of this growing patient group is warranted to maximize their benefits from TKA.

## Background

Osteoarthritis (OA) is a chronic progressive degenerative joint disorder causing considerable pain, disability, and socioeconomic costs [1]. Overall, about 7% of the world´s population is affected by OA [2]. Knee OA - the most common form of OA - accounts for approximately 360 million cases worldwide [2]. Different patient characteristics have been identified as important risk factors for OA development of which obesity is one of major concern: It has been shown that with a 5 kg/m^2^ increase in body mass the risk of knee OA increases by 35% [3].

Total knee arthroplasty (TKA) is the final management option for end-stage knee OA [4]. This complex surgery aims at the relief of pain and functional limitations, thus enhancing patients’ quality of life. Yet, objective surgical outcomes (e.g., increased joint range of motion) may not always correlate with patients’ subjective perception. Therefore, subjective patient-reported outcomes (PROs) measuring health directly from the patient [6] are of great importance [7]. Actually, about 10–20% of TKA patients with an objectively successful TKA report being disappointed with treatment outcomes [4, 6, 8]. Several different reasons for this are discussed in the literature - including the role of obesity [4–8]. However, studies investigating PROs after TKA in obese patients find conflicting results: For example, whereas some studies suggest worse functional outcomes following TKA in obese patients [9], others report similar [12] or even superior [13] outcomes in this patient group. The same is true for patient satisfaction, which has been suggested to be associated with pain relief, functional improvement [14], pre-surgical patient expectations [15, 16], (un)met expectations [14], and superior mental health [5, 17] following TKA. While some studies report similar satisfaction rates among obese versus non-obese patients (8, 18, 19], others found the opposite [6, 7].

Most studies investigating the impact of obesity on PROs following TKA only evaluate mid- to long-term outcomes [13, 20–22], while research into short-term outcomes is sparse. Yet, a better insight into short-term outcomes is strongly warranted because the greatest improvements in PROs occur during the first 3 months after surgery [23]. A better understanding of factors influencing short-term outcomes could help to reduce the relatively high dissatisfaction rates that have been witnessed among the TKA population. A recent study identified obesity as a risk factor for worse short-term functional outcome after TKA [24]. Additionally, obese patients seem to be more likely to be readmitted to the hospital shortly after TKA (25); this could be due to higher complication risks such as infection and deep vein thrombosis [26]. Also noteworthy, studies found generally lower levels of physical activity and self-efficacy in obese OA patients, [27, 28] which might be connected to lower functional gains during rehabilitation – especially in severely obese patients [29]. These factors imply that it is imperative to examine the role of obesity in an early period after TKA to further improve the quality of care.

Therefore, this study aims to investigate PROs regarding knee function as well as patients’ expectations, treatment satisfaction and psychological wellbeing among obese versus non-obese TKA patients both before surgery and shortly (i.e., 8 weeks) after TKA.

## Materials and methods

### Study design, study population and sample

This study is a secondary analysis of data retrieved via a prospective single-centre cohort study investigating knee joint function and health care services utilization in patients undergoing TKA (FInGK Study) with a pre-planned minimum sample size of 240; eventually, 241 patients were included in the study [30]. For the primary study, patients scheduled for unilateral primary or revision TKA in a specialized University Hospital in north-western Germany were consecutively recruited between December 2019 and May 2021. All patients aged 18 years and older, undergoing elective TKA, and providing sufficient cognitive ability as well as German language skills were eligible for study participation. A positive ethics vote was issued by the Ethics Committee of the University of Oldenburg (2019-064). All subjects signed an approved informed consent form before enrolment.

### Data collection

Baseline values (t0) of enrolled subjects were recorded via self-reported questionnaires upon hospital admission usually one day before surgery. Follow-up surveys were conducted two months (t1) and 12 months (t2) after TKA via postal questionnaires. For the current secondary analysis, data from baseline (t0) and two months follow-up (t1) surveys were exploratively analysed.

### Outcome measurements

Both questionnaires (t0 and t1) comprised questions on (1) quality of life and general health status, (2) pain, function, and expectations on surgery, (3) use of specific health services, and (4) sociodemographic and lifestyle factors. The t1 questionnaire additionally included questions on rehabilitation, satisfaction with the surgery, and expectation fulfilment.

The *Body Mass Index (BMI)* is defined as weight in kilograms divided by the square of body height in meters (kg/m²) [31]. It constitutes one of the most important measures of obesity [32]. For this study, information on body height and weight were obtained from hospital medical records as determined one day before surgery. Missing values (n = 4) were completed via patient self-report. Subjects were then classified as either non-obese (BMI < 30 kg/m²) or obese (BMI ≥ 30 kg/m²) according to the WHO classification [31] and analogous to previous studies [9, 12, 19, 33].

The self-administered *Western Ontario and McMasters Universities Osteoarthritis Index (WOMAC)* was used at t0 and t1 to evaluate knee OA-related pain (five items), stiffness (two items), and functional limitations (17 items). Each item included five possible response options presented as a Likert scale ranging from 0 (“none”) to 4 (“extreme”) [34]. Scores were summed up to a total WOMAC score (min-max: 0–96), with higher scores indicating higher disease burden.

Patient expectations and expectation fulfilment were assessed using the pre- and post-operative *German New Knee Society Score* (GNKSS, 2011 version) [35]. Pre-operative GNKSS expectation questions about pain relief, activities of daily living (ADL), and leisure activities were asked at t0. Each question was scored on a Likert scale ranging from 0 (“none”) to 4 (“a lot”). The results of GNKSS categories 0 (“none”), 1 (“a little”), and 2 (“somewhat”) were eventually combined and presented as one single category named “none/low”, category 3 (“moderate”) stayed unchanged, and category 4 “a lot” was renamed as “high”. At t1, subjects were also asked to indicate on a scale from 0 (“far too high”) to 4 (“far too low”) whether their expectations regarding the above-mentioned categories had been adequately met. Additionally, the t1 questionnaire contained a single question on overall surgery outcome satisfaction scored on a scale from 1 (“very good”) to 5 (“very bad”).

The *World Health Organisation-Five Well-Being Index (WHO-5)*, a widely used instrument for the generic assessment of psychological wellbeing [36], was obtained at t0 and t1. It consists of five positive statements for which respondents should indicate how well they have applied to them during the past two weeks. Five given response options from 0 (“never”) to 5 (“always”) are available. Individual scores are summed up and transformed into a total score ranging from 0 to 100, where higher scores indicate better wellbeing [36].

Level of education was classified as low, middle, or high according to the International Standard Classification of Education (ISCED) [37]. In Germany, up to nine years of scholarly education or leaving school without graduating (ISCED level 0–2B) is equivalent to having a low educational level. A medium educational level instead is equivalent to 10 years of schooling (level 2 A). Finally, a high educational level - which provides access to higher education - corresponds to 12 or 13 years of schooling (level 3 A).

### Data analyses

All descriptive data were stratified according to patients’ obesity status (BMI < 30 kg/m² vs. BMI ≥ 30 kg/m²). Descriptive statistics established means with standard deviations (SD) for continuous variables and percentages for discrete variables counts. Obese and non-obese patients were compared using Pearson´s chi-squared test and Student´s t-test for categorical variables and continuous variables, respectively. Univariable regression was used to evaluate characteristics that are associated with outcome satisfaction two months after TKA. Levels of satisfaction were dichotomized for analyses purposes. Subjects’ reporting their TKA outcome as “(very) good” were categorized as “satisfied” whereas subjects reporting “fair” and “(very) bad” TKA outcome were categorized as “dissatisfied”. The following possibly associated factors were identified via a comprehensive literature review: Sex, age, level of education, marital status, smoking status, obesity status, WOMAC, and WHO-5 baseline scores [4, 5, 17, 38–42]. Finally, all variables were included in a multivariable model. Linear regression was used to evaluate factors associated with higher WOMAC change two months after surgery. The aforementioned factors were included as covariates in the model. For logistic regression odds ratios (ORs) and for linear regression parameter estimates (β) were calculated with 95% confidence intervals (CI) and p-values. Non-overlapping 95% CIs and p-values < 0.05 were considered statistically significant.

Data analyses were performed using SPSS Statistics (Version 27, IBM, Armonk, U.S.).

## Results

### Response and baseline characteristics

During the study period, 296 ﻿elective primary or revision TKA were undertaken. Of these, 13 had to be excluded due to the following criteria: (1) already participating with their contralateral knee (n = 6), (2) language barrier (n = 6), and (3) cognitive impairment (n = 1) rendering questionnaire completion impossible. Of the remaining 283 eligible individuals, 241 (85.2%) agreed to participate. The response at t1 was 97.9% (n = 236). Drop-out reasons were (1) inability to fill in the t1 questionnaire (n = 2) and (2) refusal to further participate in the study (n = 3).

Baseline characteristics stratified by obesity status can be seen in Table 1. With 63.9%, the majority of subjects were obese. Mean BMI in the non-obese group was 9.5 points lower than in the obese group, although with 26.8 kg/m² still belonging to the overweight range (25 to < 30 kg/m^2^). Obese subjects were significantly younger than non-obese subjects at the time of TKA (67.1 vs. 70.7 years) and there were slightly fewer female subjects in the obese group. In the non-obese group, there were more current smokers and the proportion of unmarried subjects was higher. Furthermore, a low level of education was more common in the obese (54.3%) compared to the non-obese group (43.0%). Across all expectation categories, obese subjects reported less often high expectations regarding TKA, with the vastest difference concerning ADL (58.2% vs. 70.9%). Additionally, WHO-5 mean scores were significantly lower (indicating worse psychological wellbeing) among obese subjects (39.9 vs. 49.6).

**Table 1:** Baseline characteristics of the study sample in %; total and stratified by obesity status

Characteristics	Total (n = 241)	Non-obese (n = 87)	Obese (n = 154)	p-value
**BMI (mean; SD) (n = 241)**	32.9 (6.2)	26.8 (2.2)	36.3 (4.9)	-
**Age (mean; SD) (n = 241)**	**68.4 (9.4)**	**70.7 (9.6)**	**67.1 (9.0)**	**0.004**
**Sex (n = 241)**				
Female	59.8 (144)	63.2 (55)	57.8 (89)	0.409
Male	40.2 (97)	36.8 (32)	42.2 (65)	
**Smoking status (n = 240)**				
No (longer)	89.6 (215)	87.4 (76)	90.8 (139)	0.394
Current smoker	10.4 (25)	12.6 (11)	9.2 (14)	
**Marital status (n = 239)**				
Not married^§^	33.1 (79)	37.6 (32)	30.5 (47)	0.262
Married	66.9 (160)	62.4 (53)	69.5 (107)	
**Level of education (n = 237)**				
Low	50.2 (119)	43.0 (37)	54.3 (82)	0.244
Middle	32.1 (76)	37.2 (32)	29.1 (44)	
High	17.7 (42)	19.8 (17)	16.6 (25)	
**Expectations pain (n = 240)**				
None/Low	2.5 (6)	2.3 (2)	2.6 (4)	0.579
Moderate	14.6 (35)	11.5 (10)	16.3 (25)	
High	82.9 (199)	86.2 (75)	81.0 (124)	
**Expectations ADL (n = 239)**				
None/Low	7.5 (18)	3.5 (3)	9.8 (15)	0.078
Moderate	29.7 (71)	25.6 (22)	32.0 (49)	
High	62.8 (150)	70.9 (61)	58.2 (89)	
**Expectations physical activities (n = 240)**				
None/Low	17.9 (43)	13.8 (12)	20.3 (31)	0.453
Moderate	34.2 (82)	35.6 (31)	33.3 (51)	
High	47.9 (115)	50.6 (44)	46.4 (71)	
**WHO-5 (n = 233)**	**43.4 (25.0)**	**49.6 (26.2)**	**39.9 (23.6)**	**0.004**

n = Number of subjects; SD = Standard deviation; BMI = Body mass index; ADL = Activities of Daily Living;

WHO-5 = WHO-Five Well-Being Index; § Single/separated/divorced/widowed; Sig. findings in bold.

### WOMAC scores, expectation fulfilment, and satisfaction

Table 2 shows WOMAC scores at t0 and t1 as well as WOMAC change scores, expectation fulfilment, and levels of satisfaction among obese versus non-obese subjects. Mean WOMAC scores were significantly higher (indicating higher disease burden) in the obese group at t0 (52.6 vs. 46.8) and t1 (32.3 vs. 24.4), respectively. However, there was no significant difference in between-groups WOMAC change scores at t1. Furthermore, expectations fulfilment and satisfaction at t1 did not differ significantly between obese versus non-obese subjects. Comparing the different expectations categories, expectations were most often met or even exceeded regarding pain relief (64.5%), with similar distribution among obese and non-obese subjects. However, regarding the two other expectations categories, (non-significant) between-groups differences could be detected: 60.6% of obese and 69.3% of non-obese subjects reported fulfilled or even exceeded expectations regarding ADL. 47.9% of obese and 57.9% of non-obese subjects reported fulfilled or exceeded expectations regarding physical activities. Overall, 65.8% of obese and 75.9% of non-obese study participants were (very) satisfied with their outcomes two months after TKA.

**Table 2:** WOMAC scores, expectations, and outcome satisfaction at baseline and 2 months post-TKA; total and stratified by obesity status

Characteristics	Total (n = 241)	Non-obese (n = 87)	Obese (n = 154)	p-value
**WOMAC**				
Baseline; mean (SD) (n = 230)	**50.5 (14.6)**	**46.8 (15.2)**	**52.6 (13.9)**	**0.004**
2 Months FU; mean (SD) (n = 220)	**29.5 (17.5)**	**24.4 (15.3)**	**32.3 (18.0)**	**0.001**
Change between baseline and 2 months FU; mean (SD) (n = 214)	21.1 (18.6)	23.2 (17.0)	19.9 (19.4)	0.221
**Expectations pain (n = 231)**				
(Far) too high	35.5 (82)	34.6 (28)	36.0 (54)	0.932
Just right	41.6 (96)	43.2 (35)	40.7 (61)	
(Far) too low	22.9 (53)	22.2 (18)	23.3 (35)	
**Expectations ADL (n = 225)**				
(Far) too high	36.4 (82)	30.8 (24)	39.5 (58)	0.361
Just right	43.1 (97)	44.9 (35)	42.2 (62)	
(Far) too low	20.4 (46)	24.4 (19)	18.4 (27)	
**Expectations physical activities (n = 220)**				
(Far) too high	48.6 (107)	42.1 (32)	52.1 (75)	0.369
Just right	35.9 (79)	40.8 (31)	33.3 (48)	
(Far) too low	15.5 (34)	17.1 (13)	14.6 (21)	
**Satisfaction with result of surgery (n = 232)**				
(Very) good	69.4 (161)	75.9 (63)	65.8 (98)	0.232
Fair	21.6 (50)	15.7 (13)	24.8 (37)	
(Very) bad	9.1 (21)	8.4 (7)	9.4 (14)	

n = Number of subjects; SD = Standard deviation; WOMAC = Western Ontario and McMaster Universities Osteoarthritis Index; TKA = Total Knee Arthroplasty; ADL = Activities of daily life; FU = Follow-up; Sig. findings in bold

### Factors associated with TKA satisfaction

Smoking status, WOMAC, and WHO-5 baseline scores were significant predictors of TKA satisfaction in univariate analyses. However, in the multivariable logistic regression model, smoking status was the only significant predictor of TKA satisfaction (Table 3). Non-smokers had a higher chance (OR: 3.56; 95% CI: 1.21–10.45) of being satisfied with the surgery outcomes.

**Table 3:** Factors associated with TKA satisfaction two months after surgery: results from univariable and multivariable (n = 211) logistic regression analyses

Characteristics	Reference	Univariable analysis OR (95% CI)	Multivariable analysis OR (95% CI)
**Sex**			
Female	Male	0.99 (0.56–1.75)	1.07 (0.55–2.10)
**Age**	Per year	1.03 (1.00-1.06)	1.02 (0.98–1.05)
**Level of education**			
High	Low	0.90 (0.42–1.91)	0.88 (0.37–2.06)
Middle	Low	1.11 (0.58–2.12)	1.06 (0.52–2.20)
**Marital status**			
Married	Not married^§^	0.92 (0.50–1.69)	0.91 (0.44–1.85)
**Smoking status**			
No (longer)	Current smoker	**3.76 (1.53–9.27)**	**3.56 (1.21–10.45)**
**Obesity status**			
Non-obese	Obese	1.64 (0.89–3.01)	1.68 (0.83–3.41)
**WOMAC baseline score**	Per 1 unit	0.98 (0.96-1.00)	0.99 (0.97–1.02)
**WHO-5 baseline score**	Per 1 unit	1.02 (1.00-1.03)	1.01 (1.00-1.03)

TKA = Total Knee Arthroplasty; n = Number of subjects; OR = Odds Ratio; CI = Confidence Interval; WOMAC = Western Ontario and McMaster Universities Osteoarthritis Index; WHO-5 = WHO-Five Well-Being Index; ^§^Single/separated/divorced/widowed; Sig. findings in bold

### Factors associated with higher WOMAC change scores

Linear regression analysis showed that an increase of the WOMAC baseline score by 1 point (indicating higher disease burden) was associated with an increase of the WOMAC change score by 0.76 points (95% CI: 0.57–0.93). Furthermore, being non-obese was associated with a 6.33-points higher WOMAC change score (95% CI: 1.55–11.12) compared to being obese (Table 4). Sex, age, level of education, marital status, smoking status, and WHO-5 baseline scores were not associated with WOMAC change scores two months after TKA.

**Table 4:** Factors associated with higher WOMAC change 2 months after TKA: results from linear regression analyses (n = 204)

Characteristics	β	95% CI	p
**Intercept**	-37.75	-59.06-(-16.44)	0.001
**Sex**			
Female	0.32	-4.48-5.11	0.897
**Age**	0.09	-0.17-0.35	0.495
**Level of education**			
High	-0.87	-6.96-5.22	0.778
Middle	0.47	-4.62-5.56	0.856
**Marital status**			
Married	2.88	-2.06-7.84	0.251
**Smoking status**			
No (longer)	6.81	-1.22-14.83	0.096
**Obesity status**			
Non-obese	**6.33**	**1.55–11.12**	**0.010**
**WOMAC baseline score**	**0.76**	**0.57–0.93**	**0.000**
**WHO-5 baseline score**	0.10	-0.01-0.21	0.068

n = Number of subjects; CI = Confidence Interval; Std. Error = Standard Error; TKA = Total Knee Arthroplasty; WOMAC = Western Ontario and McMaster Universities Osteoarthritis Index; WHO-5 = WHO-Five Well-Being Index; Sig. findings in bold

## Discussion

The results of this explorative study suggest that obese subjects’ baseline WOMAC scores (i.e., pain, stiffness, and subjective knee joint function) are inferior compared to non-obese subjects. Moreover, obese subjects experience higher postoperative WOMAC scores (indicating higher disease burden) versus non-obese subjects. In the current study, obese subjects’ preoperative expectations were slightly (non-significantly) lower compared to those of non-obese subjects. Being non-obese and experiencing higher disease burden at baseline were predictive of higher WOMAC change scores at two months after TKA. Obesity status was, however, not associated with TKA satisfaction. The only significant predictor of higher satisfaction found in this study was smoking status, with non-smoking leading to significantly higher chances of being satisfied with TKA outcome two months after surgery.

### Preoperative expectations

The level of TKA outcome expectations was generally high in both obese and non-obese subjects. This is also reflected by the proportion of subjects reporting two months postoperatively that their pre-operative expectations had been too high. For example, 52.1% of obese and 42.1% of non-obese subjects stated that their expectations regarding physical activities had been (far) too high. Research comparing TKA outcome expectations specifically among obese versus non-obese patients is still scarce. However, available studies suggest that patients with poor preoperative functional and pain status nevertheless have comparably high TKA outcome expectations versus patients with a notable better preoperative status [32]. These findings are in line with the current study’s results, where inferior preoperative function and pain scores of obese subjects did not significantly diminish their level of expectations regarding TKA. Insufficient preoperative discussion with patients about the association of personal risk factors (e.g., obesity and/or inferior preoperative functional status) and poorer TKA outcomes may partly explain the relatively high dissatisfaction rates following TKA. This consideration is supported by Tolk et al. (2021). These authors were able to show that better patient education can have a regulatory impact on TKA-patients’ frequently inflated outcome expectations and consequently lead to higher post-operative satisfaction [16].

### Patient satisfaction

According to this study’s results, it seems that two months after surgery obese and non-obese patients are similarly satisfied with their TKA outcomes. This finding is in line with the results of Van Onsem et al. [40]. These authors evaluated patient satisfaction three months after TKA and did also not observe an association between satisfaction with the surgery and BMI. Other studies examining TKA satisfaction in the long term (i.e., 12- or 24-months following surgery) suggest conflicting results [6–8, 18]. While some authors report similar satisfaction rates among obese versus non-obese patients [8, 18], others found obese patients to be less satisfied following their TKA [6, 7]. These opposing findings may be explained by the fact that patient satisfaction is a complex concept with a multitude of potential determinants. These range from patient-related factors such as obesity, age, sex and education to health-service provider related factors such as access to care and interpersonal care quality [43]. In addition, a globally accepted definition and theoretical framework of patient satisfaction have not yet been developed [44]. Consequently, measurement instruments of patient satisfaction following TKA vary a lot across studies. Moreover, statistical analyses differ in their consideration of possible confounders [6–8, 18]. Such variability in measurement and statistical analyses hardens the comparison of related studies, thus hampering the interpretation of single factors’ (e.g., obesity) possible impact on patient outcome satisfaction.

Smoking status, which was a significant predictor of patient satisfaction in the current analysis, has rarely been assessed so far in this context. Only two studies examining the impact of smoking on patient satisfaction were found [45, 46]. Rissolio et al. assessed long-term outcomes of TKA (minimum follow-up: 24 months) and reported no association between smoking and surgery outcome satisfaction [45]. Halawi et al., however, who assessed patient satisfaction six and 12 months after TKA, found smokers to be less satisfied versus non-smokers (82% vs. 89%, p = 0.052) – independent of their psychological health status [46]. A possible reason for these opposing results could be that smoking increases the risk for periprosthetic joint infections, surgical complications, and wound complications, which may have a larger impact in the short- and medium-term [46] than in the long term [45].

### WOMAC change scores

The linear regression model used in the current study suggests that a higher WOMAC score (i.e., higher preoperative disease burden) and being non-obese are predictive of higher WOMAC change scores two months after TKA. On average, obese patients showed a higher burden of disease - expressed in higher preoperative WOMAC scores - and thus a predictive factor for more WOMAC improvement. Apart from that, however, this study’s regression analysis findings imply that obesity is an independent risk factor for smaller WOMAC change scores, i.e., less improvement in pain, stiffness, and functional limitations compared to non-obese patients. Important to keep in mind regarding this finding is the question of clinical significance. Studies investigating minimal clinically important differences (MCID) for WOMAC score changes after TKA report values of 10–15 points for the total WOMAC score [47–49]. Although MCID can vary substantially depending on timeframe, study population, and calculation methods [50, 51], it cannot be ruled out that the smaller WOMAC change scores found among obese patients in the current study may have lacked clinically importance when considered in isolation. Nevertheless, the six points difference in WOMAC change scores attributable to obesity status may still have important implications for the treatment of obese patients. Since obesity often represents just one of several risk factors for a worse postoperative outcome after TKA, obesity can become a relevant point to take into account when combined with other risk factors. This aspect may be worth considering when organizing the postoperative (inpatient) rehabilitation plan, the intensity of physiotherapeutic exercise and other supportive postoperative care, for example.

Papakostidou et al. [12] conducted a similar analysis to the current study for WOMAC change scores at 12 months following surgery. They found higher WOMAC baseline function and pain scores but not obesity status to be predictive factors of higher WOMAC change scores. This contradiction to the results of the current study could be attributable to a possibly more difficult rehabilitation process in obese patients. Research results suggest that obese patients’ rehabilitation progress after TKA is lengthier and less efficient compared to non-obese patients [29]. This would probably impact the short-term outcomes as assessed in the current study, but not necessarily the WOMAC change scores after 12 months as evaluated by Papakostidou et al. [12].

In a recent study, Giesinger et al. [52] compared WOMAC change scores between normal weight versus obese patients 12 months after TKA. They found significantly larger WOMAC score improvements in obese patients (BMI: 30 to < 40 kg/m²) compared to normal weight patients (BMI: <25 kg/m²); however, no significant WOMAC improvement differences were detected between obese patients and the large group of pre-obese patients (BMI: 25 to < 30 kg/m²; 36.9% of the study population). A possible interpretation might be that obese patients who present with preoperatively more severe pain, stiffness, and functional limitations, i.e., higher WOMAC baseline scores, have theoretically more room for improvement than non-obese patients who experience already lower preoperative WOMAC scores. Additionally, the study of Giesinger et al. comprised only 14.8% severely obese subjects (BMI: ≥35 kg/m²) as opposed to 33.6% in the current investigation. Nevertheless, these results all show that TKA is capable of effectively improving WOMAC scores among obese and non-obese patients, although obese patients may experience lengthier short-term recovery.

### Strengths and limitations

This study´s results must be interpreted in line with its strengths and limitations. Strengths of this study include the prospective design as well as the combination of outcomes, which up to this point have rarely been assessed simultaneously. Short-term satisfaction and subjective functional outcomes following TKA were evaluated jointly with patients´ expectations and mental health status specifically in obese patients. To our knowledge, this is the first study to provide new insights into the role of obesity in early PRO after TKA. Noteworthy are also the high study participation (85.2%) and that nearly all patients participated during follow-up. This significantly diminishes the risk of selection bias which may occur with a large number of non-responders. However, there are also a few limitations to address: First, patient recruitment for this study took place during the COVID-19 pandemic. This might have negatively impacted rehabilitation programs after TKA and consequently short-term satisfaction and functional outcomes. However, there is currently no indication that this aspect could have impacted rehabilitation of obese versus non-obese patients differently. Second, the data analyzed in this explorative study were retrieved from a prospective cohort study investigating pain and function as well as the utilization of health care services in patients undergoing/following TKA. As our sample size of 240 patients was planned for the primary study using the endpoint physiotherapy use within 12 months following TKA, the possibility of lack of sufficient statistical power in this secondary analysis focusing on TKA function and post-operative satisfaction of obese vs. non-obese patients cannot be fully ruled out. Third, due to the relatively small sample size we only compared two patient groups instead of further dividing obese patients into subgroups according to their WHO level of obesity. Yet, this could have been useful for a more nuanced assessment of the effect of obesity on PRO following TKA. Nevertheless, as short-term PRO of obese patients following TKA have not frequently been evaluated and, thus, this study intended to gain some initial insight into the topic, it seemed sufficient to employ such a rougher classification, which also has been used previously [9, 12, 19, 33]. Furthermore, using a classification similar to previous authors helps to compare the current results to the findings of other studies. Fourth, this study’s data were obtained from a single specialized orthopaedic department. Thus, the results may not apply to smaller, less specialized hospitals conducting TKA surgery. However, most studies on TKA outcomes are conducted at university hospitals so this fact in any case aids in placing the presented results into the context of current research.

## Conclusion

The current study showed inferior pre- and postoperative WOMAC scores among obese patients compared to non-obese patients. Being non-obese and experiencing higher preoperative WOMAC scores (indicating higher burden of disease) were predictive of higher WOMAC change scores after two months. The only significant predictor for TKA satisfaction after two months found in this study was non-smoking. However, as the current study incorporated a secondary analysis and followed an explorative approach, more research on TKA rehabilitation measures for obese patients especially in the short-term is needed to establish conclusive findings and thus, maximize these patient group’s benefits from TKA.

## Data Availability

The datasets generated and/or analysed during the current study are available from the corresponding author on reasonable request. The data are not publicly available due to privacy reasons as the participants did not consent to open publication of the databases when joining the study.

## Abbreviations

ADL: Activities of Daily Living
BMI: Body Mass Index
CI: Confidence Interval
GNKSS: German New Knee Society Score
ISCED: International Standard Classification of Education
MCID: Minimal clinically important difference
OA: Osteoarthritis
OR: Odds Ratio
PRO: Patient-Reported Outcomes
SD: Standard Deviation
TKA: Total Knee Arthroplasty
WHO-5: World Health Organisation-Five Well-Being Index
WOMAC: Western Ontario and McMasters Universities Osteoarthritis Index
